# 1269. Infection, Clinical Syndromes and Antimicrobial Resistance by *Aeromonas* species: 13-Year Experience with an Emerging Pathogen at a Tertiary Care Center

**DOI:** 10.1093/ofid/ofab466.1461

**Published:** 2021-12-04

**Authors:** Roberto Pineda-Reyes, Joseph Orndorff, David Reynoso

**Affiliations:** University of Texas Medical Branch, Galveston, Texas

## Abstract

**Background:**

*Aeromonas* spp. are emerging pathogens that cause a wide breadth of clinical syndromes, ranging from acute gastroenteritis to skin and soft tissue infections, sepsis, and “flesh-eating” necrotizing fasciitis. Aeromonads have been associated with natural disasters and have predominance in estuarine ecosystems, generating a negative impact on the fishing industry and aquaculture, as well as morbidity and mortality in human populations at risk. Antimicrobial resistance patterns differ by geographic locations worldwide, and studies to guide the therapy in the era of multidrug resistance are lacking in the US.

**Methods:**

A retrospective case series was designed to chart review all adult subjects who had culture proven *Aeromonas* spp. infections during the period 2008-2020. Demographic data, water exposure, clinical syndromes on presentation, origin (community-acquired vs. nosocomial) and severity of infection, antibiograms, empirical antibiotics, time-to-appropriate therapy, and treatment outcomes were collected.

**Results:**

Eighty-two subjects were included in the analysis. Demographic and clinical data is summarized in Table 1. Near 20% individuals had water exposure, including 53% of those with traumatic wound infections. Skin and soft tissue infection (including traumatic and surgical wound infections) was the most frequent clinical syndrome (51.2%). Sepsis was present on admission in 33% inpatients. Appropriate antibiotics were instituted in a median of 2 days (IQR=1-5), and the most prescribed empiric agents were piperacillin-tazobactam (48%) and meropenem (13.3%). Most isolates were susceptible to cefepime (70/71, 98.6%), levofloxacin (72/78, 92.3%) and TMP-SMX (69/78, 88.5%). Resistance to meropenem was reported in 18/31 isolates (58.1%) after 2015. Treatment failure was identified in 32.3% cases.

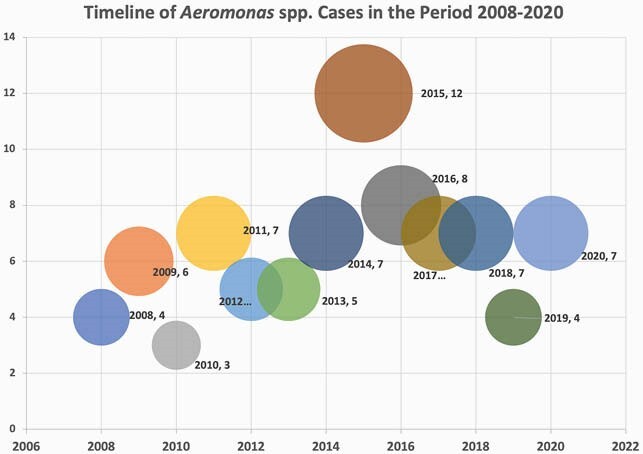

Most cases (55%) were encountered during the months of spring and summer, which have warmer temperatures and seasonal heavy rains. Tropical storms caused significant flooding in the Galveston Bay area and Southeast Texas during the summer of 2015, which interestingly coincides with the high number of cases. However, following Hurricane Ike in 2008 or Hurricane Harvey in 2017, the number of cases did not significantly increase.

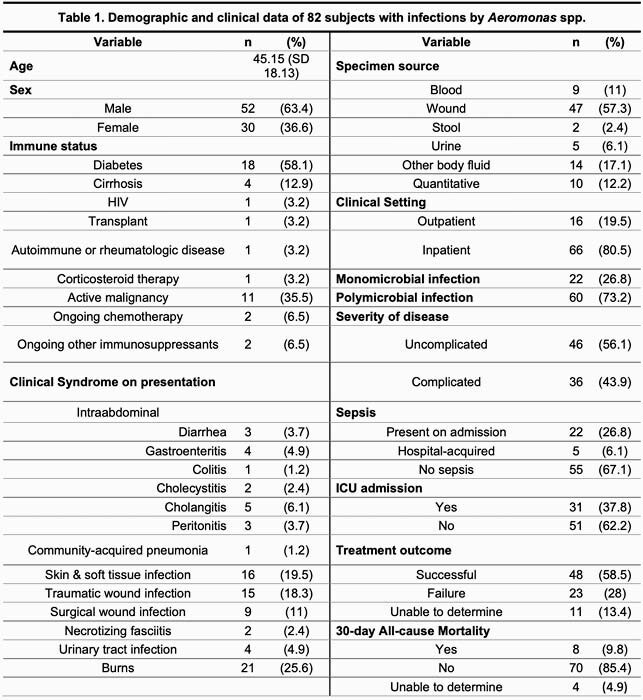

**Conclusion:**

Aeromonads are emerging pathogens that cause mainly intraabdominal and skin and soft tissue infections. Their incidence is seasonal (55% cases in spring and summer) and it is associated with water exposure in more than half of those with traumatic wound infections. In subjects with specific risk factors, the use of carbapenem-sparing strategies, such as 3^rd^ or 4^th^ generation cephalosporins, fluoroquinolones or TMP-SMX, may improve outcomes.

**Disclosures:**

**All Authors**: No reported disclosures

